# Toward a holistic view of orchard ecosystem dynamics: A comprehensive review of the multiple factors governing development or suppression of apple replant disease

**DOI:** 10.3389/fmicb.2022.949404

**Published:** 2022-07-25

**Authors:** Tracey S. Somera, Mark Mazzola

**Affiliations:** ^1^USDA, Agricultural Research Service, Wenatchee, WA, United States; ^2^Department of Plant Pathology, Stellenbosch University, Stellenbosch, South Africa

**Keywords:** apple replant disease, replant pathogen complex, orchard soil microbiome, soil amendment, rootstock genotype, phloridzin

## Abstract

Replant diseases are a common occurrence in perennial cropping systems. In apple, progress toward the development of a universally effective disease management strategy, beyond the use of broad-spectrum soil fumigants, is impeded by inconsistencies in defining replant disease etiology. A preponderance of evidence attributes apple replant disease to plant-induced changes in the soil microbiome including the proliferation of soilborne plant pathogens. Findings from alternative studies suggest that the contribution of abiotic factors, such as the accumulation of phenolic detritus from previous orchard plantings, may play a part as well. Engineering of the resident soil microbiome using resource-based strategies is demonstrating potential to limit activity of replant pathogens and improve productivity in newly established orchards. An understanding of factors promoting the assembly of a disease-suppressive soil microbiome along with consideration of host factors that confer disease tolerance or resistance is imperative to the developing a more holistic view of orchard ecosystem dynamics. Here, we review the literature concerning the transition of orchard soil from a healthy state to a replant disease-conducive state. Included in the scope of this review are studies on the influence of soil type and geography on the apple replant pathogen complex. Furthermore, several tolerance and innate resistance mechanisms that have been described in apple to date, including the role of root chemistry/exudates are discussed. Finally, the interplay between apple rootstock genotype and key resource-based strategies which have been shown to “reshape” the plant holobiont in favor of a more prophylactic or disease-suppressive state is highlighted.

## What is apple replant disease?

Apple replant disease (ARD) occurs when plant-induced changes to the soil microbiome promote the infestation of multiple host-specific, soilborne pathogens (fungal, oomycete, and nematode). Over time, the soil becomes a reservoir of pathogens causing diminished productivity of established trees and impeded establishment of new plantings (of the same or closely related species) on the same site. Although the disease is known to affect several perennial fruit and nut tree crops (e.g., pear, peach, cherry, walnut; Traquiar, 1984), this review will focus primarily on apple replant disease, a phenomenon which has been observed in all apple-producing areas worldwide including the North America, Europe, Africa, the Middle East, China, and Australasia (Hoestra, 1968; Mai and Abawai, 1981; Dullahide et al., 1994; Tewoldemedhin et al., 2011c; Xiang et al., 2021a).

Although the level of ARD severity can vary considerably both within and between orchards, symptoms typically include root browning/blackening, less extensive lateral branching and loss of fine feeder-roots owing to colonization and destruction of the root cortex by multiple pathogens (Caruso et al., 1989; Grunewaldt-Stöcker et al., 2019). As a result, ARD-affected root systems are smaller and take up resources from the soil less effectively than plants/trees growing in non-ARD soil (Mai et al., 1994). Disruption of root system structure and function results in growth and yield reductions, which can be especially severe in young apple trees and may even result in tree death. Physiological stress responses to ARD include increased concentrations of total phenolic compounds in roots (Henfrey et al., 2015; Kanfra et al., 2018) and elevated antioxidant capacity in leaves (Henfrey et al., 2015).

## Apple replant disease etiology

Apple replant disease is caused by specific types of microorganisms which act synergistically. In other words, the disease itself is the combined effect of what happens when several pathogenic soilborne microorganisms act together, rather than individually (i.e., as single isolates; Braun, 1991; Mazzola, 1998; Tewoldemedhin et al., 2011c). Although the relative proportion of pathogens may differ among sites, the key players generally remain the same (at least at the genus level). To date, the replant disease complex has been found to be the result of 3 different groups of organisms from three different kingdoms of life: Kingdom Animalia (nematodes), Kingdom Fungi (fungi), and Kingdom Chromista (oomycetes); organisms also known as water molds.

We know, without a doubt, that ARD is a biological phenomenon because soil fumigation, steam pasteurization, or soil sterilization with gamma radiation leads to restored, regular plant growth. Recognition of the effectiveness of soil fumigation to control replant disease in fruit trees began in the 1950s (Koch, 1955). Around this time, the role of root-lesion nematodes (particularly *Pratylenchus* spp.) in the development of apple replant disease and other pome fruit was becoming clear in many parts of the world including North America (Parker and Mai, 1956), Northwestern Europe (Hoestra and Oostenbrink, 1962), and Australasia (Goss, 1961; Egunjobi, 1968).

Root lesion nematodes can limit growth of apple independently but perhaps more importantly create wounds which serve as points of entry for various fungal and oomycete pathogens. *Pratylenchus penetrans* is the primary nematode species of concern in North America and throughout the world. A notable exception is India. To date, *P. penetrans* has not been reported to infect apple trees in any of the growing regions of that country (Khan et al., 2016). It should also be noted that other species of *Pratylenchus* have been implicated in ARD depending on the geographic region (Colbran, 1953; Knoetze et al., 2021). For example, *P. coffeae* was shown to be a significant factor contributing to disease development in the Granite Belt region of Australia (Colbran, 1953). In this study, *P. coffeae* was isolated from apple roots growing in ARD-conducive soil and shown to re-infect the roots of apple seedlings. Of late, culture-based analyses of potential disease-causing agents in which Koch’s postulates are satisfied like this one, however, have largely been replaced by sequence-based analyses of microbial community composition without attention to function.

Sequence-based studies aiming to understand the cause of ARD are often limited to DNA-based methodology, but the mere detection of a particular organism in orchard soil or the roots of ARD-affected apple trees does not prove the species is pathogenic to apple. The vast majority of these studies have focused on microbial community composition in bulk or rhizosphere soil, yet no attempt is made to assess the abundance of known pathogens in root tissue. Consequently, in many studies where a particular microbe is said to be causing ARD, there is no factual basis for the connection (Franke-Whittle et al., 2015; Jiang et al., 2017; Kanfra et al., 2018; Xiang et al., 2021a). Studies in which causal agents are inferred on sequence data alone may hamper the ability of the research community to clearly identify causative agents and produce confusion or debatable outcomes lasting for decades that impede progress toward developing management solutions. Further contributing to the confusion is the increased focus on publication as a measure of academic productivity, resulting in more studies with questionable aspects and/or conclusions.

Early on, treatment of replant soil with broad-spectrum biocides (e.g., chloropicrin) was found to result in a greater increases in growth and yield than when fumigants which target nematodes were used alone (Pitcher et al., 1966). Although this supported an awareness that ARD was caused by a variety of microorganisms likely working in association, a more complete understanding of the disease etiology, in terms of the specific fungi and oomycetes contributing to the disease, was not substantively investigated until the late 1970s–early 1980s. Initially, this was largely through the work of Jaffee et al. in New York State (Jaffee, 1981; Mai and Abawai, 1981; Jaffee et al., 1982) and Sewell in England (Sewell, 1981). Many studies which followed led to species-level identifications in which causal agents were isolated from ARD-affected soils and/or roots and shown capable of re-infecting apple (e.g., Braun, 1991, 1995; Mazzola, 1998; Mazzola et al., 2002). For example, Mazzola showed that specific fungi significantly contributing to the disease complex in Washington State included *Cylindrocarpon destructans* (*Ilyonectria robusta*) and *Rhizoctonia solani* AG 5 (but not *Fusarium* spp., although they were consistently recovered/isolated from diseased apple roots) as well as the oomycetes *Pythium ultimum*, *Pythium sylvaticum* and *Phytopthora cactorum*. In addition, isolates of *P. intermedium*, *P. irregulare* and *P. heterothallicum* obtained from apple roots were also shown to be highly virulent toward apple in Washington state (Mazzola et al., 2002). In the Mazzola et al. (2002) study, a large diversity of non-pathogenic *Pythium* spp. was also recovered from apple roots, some of which (if allowed to colonize the apple root system first) were even shown to enhance apple growth and/or inhibit root infection by the pathogenic strains in artificially infested soils. In both the Mazzola (1998) and the Mazzola et al. (2002) studies, the presence and/or dominance of these components in root tissue varied across a variety of orchard locations.

As previously mentioned, the relative contribution of any particular member of the pathogen complex to disease development/severity has been shown to vary among geographic regions, between orchards within the same region, and even between sites (Manici et al., 2018) or years within the same orchard location (Mazzola, 1998 vs. Wang and Mazzola, 2019a). Hence, although replant disease persists over time, temporal and/or seasonal fluctuations in population densities of different replant pathogens contradict this apparent stability. For example, nematode levels in soil and roots are largely affected by seasonal cycles and the presence of ARD or eventual nematode root populations does not always correlate with high soil densities of plant parasitic nematodes. For instance, in commercial scale trials conducted at three orchards (Dupont et al., 2021), pre-plant population of *P. penetrans* ranged from 0 to 9 per 250 g soil but root densities ranged from 142 to 997 g^−1^ at the end of the first growing season.

*Pratylenchus* spp. are migratory endoparasites which spend the majority of their life-cycle inside plant roots and their activity coincides with the active growth of apple roots. Therefore, as noted above, it is important to recognize that low *Pratylenchus* densities in orchard soil do not necessarily indicate low densities in roots planted into that soil. Soil population densities are typically highest in late autumn, when nematodes return to the soil as trees enter dormancy. During early spring, when soil temperatures generally remain below 21°C, nematode populations in soil may be less active due to overwintering (Castillo and Vovlas, 2007). Hence, failure to recover *P. penetrans* from roots of apple seedlings cultivated in replant soil may be related to when the soil was collected. For example, in a greenhouse study from Northern Italy in which *P. penetrans* was found to play an insignificant role in ARD, orchard soil used in the experiment was collected in early May (Manici et al., 2018). Instead, bi-nucleate *Rhizoctonia* spp. and *Cylindrocarpon*-like fungi were identified as being the dominant fungal genera infecting root tissue from symptomatic trees. In that study it was also noted that one strain of *Dactylonectria torrensis* and one strain of *I. robusta* significantly inhibited the growth of M9 plantlets relative to the uninoculated control.

So far, no bacteria or viruses have been *definitively* identified as causal agents of the replant disease complex. Early studies implicated Actinomycete-like organisms as causative agents of ARD (Westcott et al., 1987; Otto and Winkler, 1993). In these initial studies, microscopic observations of apple seedling rootlets cultivated in replant soil contained hyphal filaments “similar in size” (~ 1μm) to those of Actinomycetes. To date, however, no pathogenic actinomycetes have been isolated from infected root tissue and shown to fulfill Koch’s postulates. In a study by Utkhede et al. (1992), high densities (10^7^–10^8^ CFU per ml of soil) of single isolates of *Bacillus subtilis* significantly reduced the growth of apple seedlings in sterile replant soil, relative to an uninoculated control. *Total* bacterial density in bulk soil typically ranges from 10^7^–10^9^ cells g^−1^ soil. For this reason, it is important that pathogenicity assays designed to identify the causal agents of ARD be conducted in non-replant soil using plausible inoculum concentrations.

## The case against the build-up of phenolic compounds in soil

Findings from alternative studies suggest that the contribution of abiotic factors, such as the accumulation of phenolic detritus from previous orchard plantings, may play a role in apple replant disease development as well. Phloridzin represents one of the most abundant phenolic compounds found in apple roots (Emmett et al., 2014; Nicola et al., 2016), leaves, bark, and buds (Adamcová et al., 2022). Emmett et al. (2014) found that the quantity of phloridzin increases with root order and may reach concentrations of > 10% root dry weight in higher order roots (3rd and 4th order). Because phloridzin biosynthesis represents one side-branch of the phenylpropanoid pathway, alterations in phloridzin biosynthesis can directly affect the metabolic flux of other compounds within this pathway, including lignin and salicylic acid (SA; Zhou et al., 2019). Therefore, in addition to being a key component of the apple antioxidant system, alterations in phloridizin biosynthesis may also affect plant growth and abiotic/biotic stress responses.

Although reports designed to explore the role of phenolic compounds (esp. phloridzin) and other root exudates in mediating resistance to ARD have recently increased in number (Emmett et al., 2014; Yin et al., 2016; Leisso et al., 2017; Radl et al., 2019; Busnena et al., 2021; Xiang et al., 2021a) there are very few reputable studies demonstrating that phenolic and/or allelopathic compounds emanating from apple roots accumulate in the *soil* and *cause* the disease. One of the earliest studies providing evidence against this hypothesis involved the dilution of replant soil into steam-sterilized soil (Jaffee et al., 1982). In this study, even a small percentage of replant soil (0.01%) resulted in a significant increase in root discoloration relative to control treatments. The inability to dilute the causative factor from the soil suggested a biotic element that could reproduce was functional in the disease. In one highly cited study, the accumulation of phloridzin released by decaying apple root debris was suggested as a potential cause of ARD (Nicola et al., 2016). In this study, fine roots from M.26 rootstocks growing in replant soil were ground-up and mixed into uncultivated soil at a high concentration (20% v:v ratio). The effect of this amendment on the soil phenolic profile was assessed at a number of different time points: immediately after soil amendment/prior to planting, 3 months after soil amendment/prior to planting and upon harvest (4 months later). Levels of phloretin and phloridzin were significantly higher in “fresh” root-amended soil relative to all other treatments and whole plant fresh weight was also significantly reduced in this treatment relative to all other treatments. It is important to note, however, that in the “3 month old” root-amended soil phloretin and phlorizin degraded prior to planting and mean plant fresh weight was not significantly different from that of the control soils. These results showed that plant growth reductions were largely due to phytotoxicity from root-associated phenolic compounds in “fresh” soil (rather than from soilborne pathogens which would have been present at both timepoints). This study does not, however, provide evidence that ARD is *caused* by high levels of residual phenolic compounds in the soil prior to replanting. On the contrary, the study shows that phloretin and phlorizin degrade rapidly in orchard soil. Given that trees are commonly removed from old orchards 6 months to years prior to replanting, the role of these phenolics in ARD is highly questionable.

To date, there is no study demonstrating the persistence of phenolics in replant soil between time of orchard removal and replanting of the site as a fundamental cause of ARD. Studies which completely dismiss the biology and instead promote changes in soil chemistry as the primary cause of ARD are particularly problematic in this field of research, which unfortunately, continues to suffer from this unsubstantiated assessment. For example, in a peer-reviewed study published in PLOS One, it was concluded that differences in phenolic acid concentrations in the soil led to observed reductions in tree growth after replanting into old tree holes vs. aisle rows. However, there was no examination of the biology in the soil or roots from the different sampling positions (Yin et al., 2016); dramatic differences in composition of the soil/root microbiome have been documented between the tree hole and aisle row and are believed to contribute to observed growth differences (Rumberger et al., 2004). In addition, the sampling intensity was marginal (3 trees per orchard) and the statistical assessment relied on Duncan’s post-hoc test, a test which has long been superseded by other multiple comparison tests and is no longer recommended by most statisticians. By comparison, in a recent large-scale study, apple rhizosphere soil was sampled from 57 different orchard locations (at least 25 years old) within the Bohai Bay region of China (Xiang et al., 2021a). A significant positive linear relationship was identified between phloridzin content in rhizosphere soil (ranging from 2–27 mg/kg soil) and disease severity (Xiang et al., 2021a). Yet, again, it must be noted that higher levels of phloridzin in rhizosphere soil from apple trees experiencing greater disease pressure does not establish a causal link between soil chemical composition and disease incitement. Across the same 57 locations, disease severity (%) was defined as the difference between dry seedling biomass after growth in pasteurized soil and that of the replant soil, divided by seedling biomass from pasteurized soil × 100. As designed, this assay, then, evaluated and confirmed the biotic origin of the disease.

Multiple studies have provided evidence that a number of phenolic compounds (including phloridzin and its derivatives) are induced in apple roots in response to biotic stress due to replant conditions (Emmett et al., 2014; Henfrey et al., 2015; Balbín-Suárez et al., 2021). Thus, changes in root exudation patterns leading to the production of phenolic compounds and other secondary metabolites are likely to be a consequence of ARD, rather than a fundamental cause. This response is highly nuanced, as the composition and concentration of phenolic compounds varies with root branching order and also rootstock genotype (Emmett et al., 2014). In addition, different phenolic compounds may have different (or even contrasting) effects on the individual pathogens. For example, while phloridzin derivatives (including hydroxycinnamic acid) were negatively correlated with *Pythium irregulare* and *P. sylvaticum* DNA in apple root tissue, phloridzin was positively correlated with both *Cylindrocarpon* and *P. sylvaticum* DNA (Emmett et al., 2014). In addition, as noted above, flux modifications to the phenylpropanoid pathway (due to the production of phenolic compounds and other defense-related phytoalexins) may alter the degree of SA accumulation, ROS production and cellular damage in apple (Zhou et al., 2019). Taken together, host-plant chemical defenses occurring under replant conditions can drive the release of exudates which may mediate pathogen colonization of root tissue and/or result in autotoxicity, potentially exacerbating the disease (which is primarily a function of biology and can be controlled with soil fumigation/pasteurization).

## Transformation of the soil microbiome to a replant disease-conducive state

It is well known that the plant microbiome is a potential controller of wellness and disease. One way plants shape soil and root-associated microbial communities is *via* root exudation, in which plant-produced compounds (e.g., sugars, amino acids, organic acids, phenolic compounds, and proteins) are released into the rhizosphere environment. It is also well known, as is evidenced by differences between rhizosphere microbial communities and those of bulk soil, that microbial communities associated with plant roots reflect selection by the host plant. In addition, current research shows plant-associated microbial communities themselves may play a role in shaping the root microbiome by governing root exudate patterns *via* plant-microbe feedback. For example, a recent study utilized tomato plants growing in a split-root hydroponic set-up to show that root colonization by individual microbial strains (*Pseudomonas fluorescens* and *B. subtilis*) resulted in specific changes to the root-excreted metabolome at both local and distal locations (leaf and root tissue on the other side of the split-root set-up; Korenblum et al., 2020). This means root exudation patterns may change depending on the biotic context, thereby altering existing plant-microbe and microbe-microbe interactions. Progressive modifications to the rhizosphere environment can lead to successional changes that are compositionally and functionally very different from previous states in this way. The process of anaerobic soil disinfestation (ASD) provides an excellent example of how succession in soil microbial communities is coordinated through time as specific groups of microorganisms alter the existing environment and pave the way for new microbial groups. In a study by Hewavitharana et al. (2019), rapid aerobic utilization of labile carbon in rice bran substrate initiated a highly dynamic cascade of structural shifts in the soil microbiome. These biological changes were linked to sequential transformations in soil community metabolism, which were also characterized at multiple time points during the ASD process.

One of the only experiments to characterize successional changes in resident soil and rhizosphere microbial communities over time (in response to continuous planting of apple) utilized soil collected from the root zones of trees growing in orchard blocks in the first through fifth years (Mazzola, 1999). Non-cultivated (virgin soil from the same location) and 1^st^-year treatments were similar in terms of plant growth potential. Thereafter, plant biomass declined rapidly with increasing block age and correlated with a steady reduction in the relative isolation frequency of potentially antagonistic and/or ARD-suppressive bacteria in rhizosphere and/or bulk soil.

In several other studies, Actinobacteria, a chemically talented group of saprophytic bacteria (which generally specialize in degrading lignocellulosic organic matter) were shown to occur in higher relative abundance in the rhizospheres of apple cultivated in ARD-suppressive or uncultivated soil as compared to those of ARD-conducive soil from the same location (Mazzola et al., 2015; Radl et al., 2019). Using a shotgun metagenomic approach, Radl et al. found that genes involved in the anaerobic degradation of benzoate and 4-hydroxybenzoyl-CoA to benzoyl-CoA (and subsequently to Acetyl-CoA) were enriched in rhizosphere communities from M.26 rootstocks cultivated in non-replant control soil relative to replant soil. Interestingly, some of these genes are also involved in the degradation of specific phenolic compounds, of which 4-hydroxybenzoyl-CoA is an intermediate (Brackmann and Fuchs, 1993; Yadav et al., 2021). If, as it appears, microbial-induced alterations to apple root exudation patterns promote higher concentrations of phenolic compounds, then the ability to degrade phenolics would be expected to increase bacterial fitness in the apple rhizosphere. Surprisingly, however, the replant-associated microbiome was characterized by a *decrease* in this activity (Radl et al., 2019). Taken together, these data suggest that shortly after planting apple there is a phase of rapid change, in which many early colonizers carrying key metabolic traits become less abundant or are completely eliminated. If these functions are “narrow” (i.e., not found across many bacteria) and are relevant in the replant disease context (e.g., antagonistic toward replant pathogens), then the loss of specific community members (or even individual strains) can substantially impact the functional capabilities of the root-associated microbiome.

Clear differences were also observed between replant and virgin soil in the Radl et al. (2019) study in terms of bacterial life strategies (fungi represented ~ 1% of the total metagenomic reads). In the rhizosphere of apple cultivated in virgin soil, genes for resource-foraging (i.e., genes involved in nutrient sensing and uptake) were enriched, which may indicate cooperative interactions between root-associated microbiota and the plant host. In replant rhizospheres, however, genes associated with competitive microbe-microbe interactions and adverse cellular conditions, including quorum sensing, biofilm formation, antibiotic production, secretion systems, chemotaxis, and siderophore production were more abundant. Although ARD pathogens act synergistically to cause the disease, they compete for the same host. Antagonism between certain fungal/oomycete components of the ARD disease complex has been observed in multiple studies and may even involve the production of antibiotics (Manici et al., 2018). Thus, the natural transition from a disease-suppressive to a disease-conducive state is likely to involve a dramatic shift not only in the way root-associated microbes interact with the plant, but also with each other. This cooperation vs. conflict dynamic has been shown to act as a structuring force in the human gut as well (Wasielewski et al., 2016).

Studies from a diversity of ecosystems have shown that conditions resulting in increased availability of simple sugars (e.g., glucose, fructose, etc.) create a structuring force that leads to more antagonistic (and fewer mutualistic) microbe-microbe and microbe-host interactions (Wasielewski et al., 2016; Deng and Wang, 2017). This phenomenon is likely due to conflict over abundant labile carbon. In a study conducted in China, invertase activity was found to be significantly higher in both bulk and rhizosphere soil from apples planted into replant soil than in non-replant soil at the same site (Sun et al., 2014). Invertase catalyzes the hydrolysis of sucrose into glucose and fructose, and its activity is typically used as an indicator of the availability of low molecular weight sugars.

In apple, pathogenic interactions in the rhizosphere may result in simple sugars from infected root tissue becoming more available to other microbes, reducing reliance on complex carbohydrates. Deng and Wang (2017) found that lignocellulolytic groups (Actinobacteria and Bacteroidetes) were most susceptible to this type of substrate-specific antagonism. As previously mentioned, several studies have noted that saprophytic microbes including Actinobacteria and Bacteroidetes are less abundant in the rhizosphere of apple rootstocks growing in disease-conducive soil as compared to disease-suppressive or uncultivated soil. For example, in the Mazzola (1999) study, apple seedlings grown in 3rd year orchard blocks (but not 1st and 2nd) became conducive to *Rhizoctonia* root rot and their cultivable root-associated fungal communities less saprophytic in nature. Interestingly, *Pythium* and *Phytopthora* spp. were only recovered from plant roots grown in soil collected from 4th and 5th year blocks (Mazzola, 1998). Thus, over a relatively short period of time, apple plants influence the identity, abundance and function of the soil and root-associated microbes in a way that ultimately creates a highly disrupted rhizosphere-microbiome leading to plant growth inhibition (i.e., dysbiosis). Studies on black cherry trees in the wild have shown that both plant-mediated accumulation of host-specific pathogens in soil and reduced seedling survival can occur over relatively short time frames as well (Packer and Clay, 2004).

In forest ecosystems, the build-up of natural enemies in the soil is regarded as an important mechanism for regulating the spatial distribution of trees and promoting species coexistence (Connell and Slatyer, 1977). In intensive tree fruit production systems, however, the ongoing domination of host-specific pathogens in the soil leads to profound and long-lasting impacts on the future trajectory of the orchard system that are difficult to reverse. In fumigated soils, the apple rhizosphere microbiome typically reverts to that of the previous replant state within 2 years (Mazzola et al., 2015). Even orchard systems which remain fallow for years do not revert to their previous disease-suppressive state (Mazzola and Mullinix, 2005). In other words, unless alternative forms of “assistance” are provided, the ARD-conducive stable-state persists in which the root-associated microbial communities of new plantings are predetermined by and/or quickly converge (in terms of function) to that of the previous planting. Among the most effective strategies for achieving longer-term disease control are those that not only suppress the ARD pathogen complex but also “transform” the resident soil microbiome in ways that prevent pathogen reinfestation [e.g., Brassicaceae seed meal (BSM) amendments, anaerobic soil disinfestation (ASD)].

**Figure 1:**
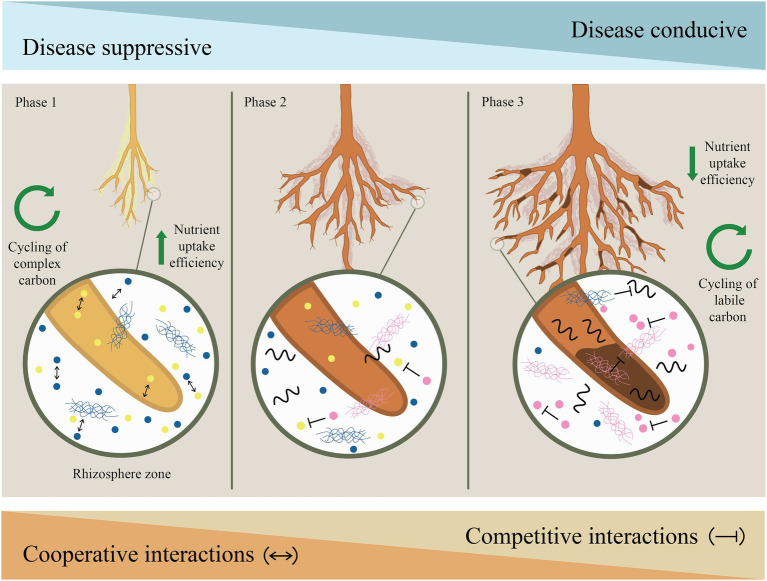
Conceptual model describing successional changes in apple root associated microbial communities leading to apple replant disease and structuring forces which drive these changes.

To date, work on apple root exudates is extremely limited and only a small number of compounds (primarily phenolic metabolites) have been considered in the context of ARD (Emmett et al., 2014; Henfrey et al., 2015; Nicola et al., 2016). However, recent metabolomic studies have expanded the foundation from which researchers have to work (Leisso et al., 2017, 2018; Busnena et al., 2021). In addition, key microbial functions contributing to disease control as well as specific environmental resources which support these functions are not entirely clear, but these are promising territories for future research. Despite the fact we lack a complete understanding of the mechanisms and temporal dynamics underlying microbial community assembly and subsequent stabilization during ARD development or suppression, a conceptual framework can be established. Here, we present a hypothetical model describing ARD-successional changes and structuring forces thought to occur upon plant establishment in soil not previously planted to apple (Figure 1).

### Phase 1. Initial tree establishment in soil not previously planted to apple

Plant roots become occupied by effective root-colonizers adapted to the local conditions including a variety of microorganisms with novel antagonistic properties toward plant pathogens (as represented by yellow dots). Saprophytic bacteria/fungi (blue dots/mesh) are dominant agents of nutrient and energy flows. Overall, community members are more efficient at nutrient uptake. Cooperative (functionally compatible) plant-microbe and microbe-microbe interactions structure the microbial communities associated with the root/rhizosphere.

### Phase 2. Transition from disease-suppressive to disease-conducive state

Root systems expand and exert greater influence on the surrounding soil. Increased production of root exudates due to plant-microbe feedback (e.g., defense-related metabolites) and/or allelochemicals that may serve as “signals” for pathogen invasion. Nematodes (black squiggles) may facilitate pathogenic fungal/oomycete (pink mesh) access to wounded tissue. Community members with competitive traits and/or specific substrate utilization profiles (pink dots) make the rhizosphere less “suitable” for growth and recruitment of keystone disease-suppressive taxa/traits (yellow dots become less abundant).

### Phase 3. Replant disease-conducive stable state

Pathogens increase in relative abundance and pathogenic fungi/oomycetes compete with each other (and with potentially beneficial microbes colonizing the root cortex). Labile sugars/carbon released from infected root systems promote competitive plant–microbe/microbe–microbe interactions. Differences in substrate availability exert negative selective pressure on saprotrophic communities. Nutrient uptake efficiency is reduced. Pathogens and community members with competitive traits (pink dots) maintain dominance over time.

## The influence of environmental factors/soil type on the pathogen complex

In all terrestrial ecosystems on the planet, soil type and/or environmental factors shape microbial community structure. Likewise, the dynamics of replant disease can be influenced by a number of environmental factors. For example, in ARD pathology, depending on location or season, different causal agents can dominate (or play a greater role) making it difficult to assess rootstock disease tolerance/susceptibility in a standardized manner. Two key environmental factors affecting the activity of ARD pathogen populations and their interactions are soil temperature and moisture. In cooler, water-logged soil conditions and/or soils that drain poorly *Phytopthora* spp. are more likely to be important contributors to disease development (Sneh and McIntosh, 1974; Traquiar, 1984). In comparison, lower soil water content may induce oospore formation (Sneh and McIntosh, 1974). Fluctuations in the abundance and species composition of *Pythium* may also occur depending on the season. For example, in a field study conducted on alfalfa, root isolation frequencies of *P. ultimum* and *P. irregulare* were significantly positively correlated with rainfall (Larkin et al., 1995). By contrast, the frequency of isolation of *P. sylvaticum* from root tissue was significantly negatively correlated with rainfall and positively correlated with soil temperature (Larkin et al., 1995). This is particularly significant because, although the Larkin et al. study was conducted with alfalfa, all three of these *Pythium* spp. are considered to be highly virulent ARD-specific pathogens. Contrasting seasonal patterns have been shown to influence the relative dominance of *Rhizoctonia* vs. *Pythium* spp. as well (Mazzola et al., 2020). Soil moisture can also be a factor in the activity and involvement of nematode populations. When topsoil dries up or conditions become adverse, *Pratylenchus* spp. have been shown to migrate to deeper layers of the soil profile, and eggs can survive even longer periods of desiccation (Mani, 1999).

Some early studies suggested that *P. penetrans* populations are higher, and therefore cause more damage, in sandy and/or coarse-textured soils (Mai and Abawai, 1981); however, this has not been well documented. That said, recently a large-scale study was conducted to assess the influence of a variety of soil characteristics on nematode population densities in apple, cherry, and grape vineyards throughout the Okanagan Valley region of British Columbia (Forge et al., 2021). This survey included soil collected from the root-zone of over 100 different sites representing five broad soil textural groups. Although levels of infestation in root tissue were not assessed, the logarithm of *Pratylenchus* population densities (primarily *P. penetrans* and *P. neglectus*) across the apple and cherry sites (n = 57) was significantly negatively correlated with the percentage of clay, which ranged from 5 to 50%. *P. penetrans* populations would, therefore, be expected to contribute less to ARD in geographic regions where fine-textured soils are more common. Nevertheless, high numbers of *P. penetrans* are frequently associated with apple roots cultivated in a variety of soil types including soils containing relatively high percentages of clay and silt (e.g., Northeastern United States) as well as in much sandier soils (e.g., Central Washington; Mai and Abawai, 1981; Mai et al., 1994; Mazzola et al., 2015; Wang and Mazzola, 2019a).

Soil organic matter (SOM) content may also be an important factor mediating replant disease severity in some geographic regions. It is difficult, however, to broadly generalize about the influence of any one soil characteristic on ARD. Data from analyses of SOM content in orchards from various locations across the globe illustrates this problem. In a recent large-scale study, which included 57 orchards in the main apple growing region of China, soil organic matter was found to be significantly negatively correlated with replant disease severity (Xiang et al., 2021a). Soil organic carbon content was <1% at 71% of the locations classified as having severe ARD, but was > 1.5% in all locations classified as having mild ARD. In Central Washington, however, orchards with relatively high soil organic matter content (2–3%) are highly conducive to ARD (Wang and Mazzola, 2019a; Mazzola et al., 2020). By notable contrast, high organic matter content (2.5–5%) resulting from long-term organic matter enrichment was reported to support the development of soil suppressiveness in the Bolzano province of Northern Italy (Mazzola and Manici, 2012). In another study from the same region, however, soils from organic orchards were more suppressive toward replant pathogens and had higher levels of total culturable fungi than those of the conventional orchards with similar soil organic matter content. This suggests management practices (rather than differences in SOM) altered the balance between pathogens and non-pathogens (Manici et al., 2003). In summary, the composition and functioning of the soil microbiome is influenced by a number of factors, none of which have been particularly useful on their own as predictors of the overall functional performance of the soil in terms of its “suppressiveness” toward replant disease. In future, long-term environmental modeling approaches which combine environmental parameters with the actual abundance and/or isolation frequencies of multiple components of the disease complex may accelerate our understanding of replant dynamics.

## How constructs of the ARD phenomenon compare based on geography

Multiple components of the ARD pathogen complex have been associated with orchard replant soil systems worldwide, including *Pratylenchus* spp., multinucleate *Rhizoctonia spp., Cylindrocarpon/Ilyonectria* spp. and several different species of *Pythium* and *Phytopthora*. Less is known about the species compositions of these groups (esp. oomycetes) and their differential virulence toward apple in any given region/area. For example, although many species of fungi (*C. destructans*) and oomycetes (*P. cactorum*, *P. irregulare*, and *P. sylvaticum*) known to be highly virulent toward apple in North America and Europe have also been definitively identified as important ARD pathogens in the main apple growing regions of South Africa, other species may be region-specific (Tewoldemedhin et al., 2011a,b). In particular, *P. vexans* and *C. macrodidymum* which have been reported to cause extensive growth reductions in apple seedlings in South Africa have rarely (Mazzola et al., 2002) or have not been reported to be associated with apple roots in North America or Europe. In comparison, *P. ultimum* and *P. intermedium*, which are frequently associated with replant disease in North America and Europe, have not yet been isolated from apple roots in South Africa (Sewell, 1981; Mazzola, 1998; Mazzola et al., 2002; Tewoldemedhin et al., 2011b).

It is also worth noting that a number of histology-based studies have utilized infected root tissue from plants grown in replant soil to specifically explore the contribution of a variety of microorganisms to ARD severity in Northern Germany (Grunewaldt-Stöcker et al., 2019, 2021; Popp et al., 2020). One study by Popp et al. (2020), combined laser microdissection or Harris Uni-Core punching with molecular techniques for the detection of endophytic fungi in highly localized regions of root tissue. In this study, multiple species of Nectriaceae including *Ilyonectria* (*Cylindrocarpon*) sp., *Dactylonectria torrensis* and *Rugonectria rugulosa* (but not *Fusarium sp*.), were more frequently identified in “symptomatic” (100% of samples analyzed) than in “symptom-free” (50% of samples analyzed) regions of root tissue. This research utilizes some of the most unique and cutting-edge approaches to studying ARD etiology in the field today. In view of the fact that many members of the Nectriaceae family (e.g., *Cylindrocarpon*/*Ilyonectria* spp., *Fusarium* spp.) contain a wide range of plant associations (from pathogenic to saprophytic), additional work will be needed to confirm which members of this Nectriaceae complex are pathogenic.

Several species within the genus *Fusarium* have been isolated from roots of apple growing in replant soils worldwide and have been suggested as possible causative agents of the disease. Numerous species of *Fusarium* were also isolated from apple roots and tested for pathogenicity in apple seedlings in the studies noted above (Mazzola, 1998; Tewoldemedhin et al., 2011b). In South Africa, only 2/10 *Fusarium* spp. isolates (*F. avenaceum* and *F. solani*) were identified as weakly virulent toward apple seedlings. Likewise, in a study conducted in Washington, it was found that among 23 *Fusarium* spp. isolates recovered from apple only a single isolate of *F. sambucinum* (from a total of 8 isolates representing this species) reduced apple seedling biomass relative to the non-inoculated control (Mazzola, 1998). In both studies, the vast majority of *Fusarium* spp. and isolates recovered from apple roots (including *F. oxysporum*) represented non-pathogenic strains. In contrast, multiple reports from China assert that *Fusarium* is the primary causative agent of ARD. In fact, the claim that *Fusarium* spp. are key pathogens associated with ARD in *other* regions of the world is a recurring inaccurate statement reported in many of these studies (Wang et al., 2018a; Xiang et al., 2021b; Liu et al., 2022). The statement regarding *Fusarium* functioning as an ARD pathogen is primarily based upon sequence-based studies in which relative abundances of *Fusarium* spp. in replant soils from the Bohai Bay and Loess Plateau region were positively correlated with disease severity in growth tests with apple seedlings (Wang et al., 2018a,b). Thus far however, the evidence in support of the role of *Fusarium* as an ARD-causing agent in China remains inconclusive. That said, limited support for this claim was recently provided in a study in which a single isolate of *F. solani* (originally isolated from apple roots growing in replant soil from the Bohai Bay region) was shown to significantly reduce plant growth of tissue cultured M.9 rootstocks relative to non-infected control plants after 1 month (1 × 10^3^ spores per ml of soil; Xiang et al., 2021b). Multiple isolates of *F. solani* recovered from apple in the Western Cape province of South Africa (Tewoldemedhin et al., 2011b) or Washington State United states (Mazzola, 1998) were found to be non-pathogenic toward apple.

To complete the discussion highlighting the potential differences in the constructs of the ARD phenomenon among geographic locations, we note a recent sequence-based study which characterized the endophytic bacterial communities associated with apple roots growing in ARD-affected soils in Germany (Mahnkopp-Dirks et al., 2021). In this study, the contribution of *Actinobacteria* (belonging to the genus *Streptomyces*) to ARD was explored in light of the multiple *Streptomycete* amplicon sequence variants (ASVs) being negatively correlated with the growth of susceptible (M.26) apple rootstock plantlets in replant soil. Interestingly, *Streptomyces* spp. ASVs with the strongest correlations had the highest sequence similarity (> 99%) to *S. turgidiscabies,* one of the few *Streptomyces* spp. known to be pathogenic toward plants. In summary, we argue that more research (i.e., further inoculation-based studies and/or pathogenicity testing) is needed to determine the contribution of *Streptomyces* and/or *Fusarium* spp. to the apple replant disease complex.

## Disease control methodologies

Pre-plant soil fumigation is the benchmark upon which all other potential control options have been measured (Covey et al., 1979; Mai and Abawai, 1981; Willett et al., 1994) and, with few exceptions (e.g., the European Union), continues to be the dominant means employed for the control of ARD. Several fumigants are utilized for this purpose, but regulatory actions often restrict the use of specific chemistry formulations. In general, formulations that possess both fungicidal and nematicidal activity have demonstrated superior levels of ARD control in field assessments. For instance, Vorlex, possessing fungicidal (20% methylisothiocyanate) and nematicidal (80% 1,3-dichloropropene) activity, provided superior ARD control relative to chloropicrin (fungicidal action only) in trials conducted in Nova Scotia (Ross et al., 1983). In a series of field trials, preplant treatment with a 1,3-dichloropropene/chloropicrin fumigant formulation significantly increased apple yields from 56 to 161% over the no treatment control (Mazzola and Mullinix, 2005; Mazzola et al., 2015; Nyoni et al., 2019; Wang and Mazzola, 2019a). However, instances have been reported in which soil fumigation with 1,3-dichloropropene/chloropicrin failed to provide significant increases in apple growth and yield on replant sites (Rumberger et al., 2004; Yao et al., 2006), and at times this has been associated with failure to diminish pathogen densities (Mazzola et al., 2015).

A diversity of alternatives to soil fumigation have been proposed and/or evaluated for the management of apple replant disease. The numerous cultural practices evaluated for ARD control include: application of mono-ammonium phosphate (Slykhuis and Li, 1985), deep ripping of soil and removal of apple roots (Braun et al., 2010), altering orchard planting pattern to avoid planting in the old tree rows (Rumberger et al., 2007), replacing soil in the tree hole with new virgin soil (Willett et al., 1994), site abandonment with establishment of the new orchard on ground not previously planted to apple, addition of organic matter to soil (e.g., compost), application of biological control agents (Utkhede et al., 2001), active manipulation of orchard soil microbiology (Mazzola et al., 2015), application of semi-selective chemical formulations targeting the pathogen complex (Nyoni et al., 2019), and the use of disease tolerant rootstocks (Auvil et al., 2011; Robinson et al., 2012). In general, these strategies have demonstrated constraints in terms of efficacy, practicality and/or economics. For instance, there are limited reports relative to the effective use of single strain microbial inoculants for the control of defined (single pathogen target) soilborne disease causal agents in annual cropping systems. Thus, successful implementation of such a treatment in combating a diverse pathogen complex in a perennial cropping system is suspect and has not been demonstrated in field trials. The semi-selective chemical treatment improved tree growth in field trials but the treatment was inconsistent and disease control was site-specific (Nyoni et al., 2019). Although utilization of ARD tolerant rootstocks does show promise in combatting the disease, enhanced performance of tolerant rootstocks relative to those identified as susceptible has been inconsistent (St Laurent et al., 2010; Wang and Mazzola, 2019a; Hewavitharana and Mazzola, 2020).

Manipulation of the orchard soil microbiome resulting in altered function and concomitant generation of biologically active chemistries has demonstrated significant promise for the control of ARD. Multiple field trials conducted at the research and commercial field scale demonstrated consistent efficacy of pre-plant Brassicaceae seed meal (BSM) amendments and anaerobic soil disinfestation (ASD) in the control of ARD in Washington State, United States. The particulars regarding the application of these treatments and effectiveness for control of ARD will be discussed below.

## Mechanisms of rootstock disease tolerance

Until recently, apple rootstock breeding programs have not explicitly targeted the development of resistance to ARD as a core program objective. Rootstock breeding historically has focused on optimization of horticultural characteristics such as vigor control and precocity (Zurawicz et al., 2011; Fazio, 2014). However, advanced rootstock selections from programs targeting these horticultural traits have subsequently been screened for tolerance or resistance to replant disease (Isutsa and Merwin, 2000; Auvil et al., 2011; Robinson et al., 2012) and material purported to possess ARD tolerance/resistance has been released commercially. Regrettably, the terms tolerance and resistance have on occasion been utilized interchangeably when characterizing the performance of apple rootstocks cultivated in orchard replant soils (Robinson et al., 2012). While tolerance refers to a plant’s ability to grow and produce despite being susceptible to pathogen infection and colonization, resistance indicates a plant’s ability to limit infection or subsequent proliferation of the pathogen. These attributes differ most notably in a mechanistic manner but also differ with regard to the effect of environment on expression of the desired response. The influence of site or environment on the function of replant disease tolerance is evident based upon the differential classification of specific rootstock genotypes in trials conducted across multiple replant sites. For instance, G.41 rootstock was classified as ARD susceptible (St Laurent et al., 2010) or “resistant” (Robinson et al., 2012) depending upon orchard soil in which the trial was conducted. Likewise, Gala/G.41 demonstrated a growth and yield response to soil fumigation equivalent to that of the highly susceptible Gala/M.26 (Wang and Mazzola, 2019a) indicating the failure of G.41 to express field tolerance to the ARD pathogen complex at the study site. Therefore, although the classification of rootstocks in terms of relative ARD tolerance is of interest and potential utility to the producer, predicting the utility of this tolerance requires on-site evaluation of rootstock germplasm prior to implementation in commercial orchards.

Manifestation of ARD in apple is believed to progress due to fine root tip attrition resulting from pathogen attack causing diminished water and nutrient absorption. The ability to rapidly replace finer root tissue appears to have a significant role in the tolerance expressed by certain rootstock genotypes (Atucha et al., 2013; Emmett et al., 2014). Tolerance to ARD was associated with anatomical differences among rootstocks with tolerant rootstocks having finer foots and a highly branched structure (Emmett et al., 2014). The tolerant rootstock genotype G.210 had higher below ground biomass and rate of new root production than the disease susceptible rootstock M.26 planted in ARD soil. It was hypothesized that the greater accumulation of below ground biomass and root length allowed the G.210 rootstock to compensate more effectively than the susceptible rootstock M.26 for root loss resulting from infection by ARD pathogens. A comparative analysis of the transcriptome from M26 and G.210 root tissue, when cultivated in replant soil, further supported the role of increased resource allocation to root production as a mechanism contributing to enhanced performance of G.210 rootstock on orchard replant sites (Wang et al., 2021). At the same time points, genes involved in cell wall organization and biogenesis were more highly expressed in G.210 than M.26, a finding which may be related to the need in G.210 to synthesize biomass components for rapidly dividing root cells. Other aspects that may be related to new biomass production included up regulation in G.210, relative to M.26, of genes involved in protein synthesis and DNA replication (Wang et al., 2021).

Apple rootstock genotypes differ in composition and activity of the rhizosphere/endophytic microbiome (Liu et al., 2018; Deakin et al., 2019; Wang and Mazzola, 2019b; Van Horn et al., 2021) and metabolome (Leisso et al., 2017, 2018) which may function in the reported tolerance of specific genotypes to the pathogen complex that incites ARD. The rhizosphere and endophytic microbial community detected in apple differed significantly among ARD susceptible and tolerant rootstocks (Van Horn et al., 2021). The relative abundance of mycorrhizal species within the Glomeraceae was significantly higher in the endophytic fungal community detected in tolerant rootstocks (G.41, G.890, and G.935) than in susceptible rootstocks (M.9 and M.26) when cultivated in replant orchard soil (Van Horn et al., 2021). Mycorrhizal colonization of plant roots can inhibit root infection by pathogens and parasites of apple (Forge et al., 2001; Graham, 2001), but pathogen suppression notably occurs in a mycorrhizae species-dependent manner (Forge et al., 2001; Gough et al., 2020). The tolerant rootstock G.890 consistently possessed the greatest abundance of OTUs designated as *Funneliformis mosseae*, a species reported to suppress apple root colonization by *Pratylenchus penetrans* (Forge et al., 2001; Gough et al., 2020). Although application of mycorrhizal inoculants in controlled environment studies mitigated the growth suppressive effects of ARD (Cavael et al., 2021), use of an inoculant, which commonly employs a single or few species, at the field scale is unlikely to supplant infection by the diverse mycorrhizal community resident to an orchard soil system.

Differential metabolic composition of apple rootstock rhizodeposits may also contribute directly or indirectly to ARD tolerance/susceptibility. Numerous triterpenoids and phenolic compounds detected in apple root rhizodeposits (Leisso et al., 2017, 2018) demonstrate antifungal activity (Cho et al., 1998; Song et al., 2011; Ma et al., 2017). A number of these metabolites were found to differ significantly in concentration between tolerant (G.935 and G.41) and susceptible (M.9 and M.26) rootstocks (Leisso et al., 2017, 2018). Among these, phloridzin was detected at significantly higher concentration in M.9 and M.26 while benzoic acid and hydroxybenzoic acid were present at a higher concentration in G.935 rhizodeposits. Phloridzin is the most extensively studied phenolic apple root exudate. A diversity of potential functions assigned to this apple metabolite include: *in vitro* antifungal activity (Shim et al., 2010), host detection signal for plant pathogens (Hoffman et al., 2009), anti-herbivore compound (Lauzon et al., 2003), promoter of apple growth (Jones, 1976), inhibitor of apple growth and a cause of replant disease (Nicola et al., 2016). However, its function as an antimicrobial agent is suspect, as addition of phloridzin amplified fungal density, had no effect on bacterial density and did not alter composition of the microbiome detected in a replant orchard soil. In addition, phloridzin demonstrated no *in vitro* inhibitory activity toward the apple root pathogens *Phytophthora cactorum*, *P. ultimum* or *R. solani* AG-5. In contrast, hydroxybenzoic acid inhibited all three microorganisms and benzoic acid inhibited growth of *P. ultimum* and *R. solani* AG-5 (Table 1).

**Table 1:** Effect of select phenolic metabolites detected in apple root exudates on *in vitro* radial growth (cm) of targeted apple root pathogens.

Treatment^z^	*Phytophthora cactorum*	*Pythium ultimum*	*Rhizoctonia solani* AG-5
Control	2.42a^y^	3.29a	6.88a
Phloridzin	2.07a	3.22a	5.73ab
Benzoic acid	0.00b	0.00c	4.49bc
4-Hydroxy-benzoic acid	1.96a	1.44b	3.80c

z Individual metabolites were added to potato dextrose agar at a rate of 0.0125 mg ml^−1^.

y Means in a column followed by the same letter are not significantly (*p* = 0.05; *n* = 5) different according to Tukey’s test.

**Figure 2:**
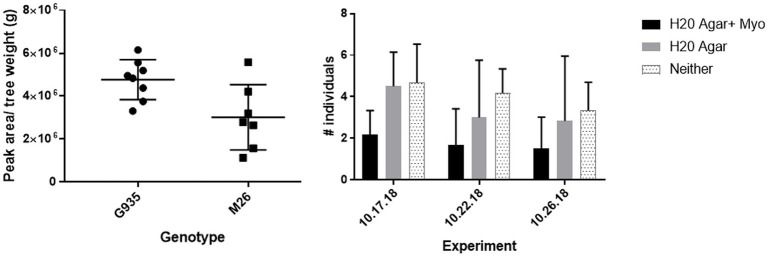
Left panel: relative quantity of myo-inositol as determined by LC–MS QTOF analysis (Leisso et al., 2017) in root exudates of micro-propagated G.935 and M.26 plantlets cultivated in root elongation medium (Yepes and Aldwinckle, 1994). Quantity of myo-inositol detected was significantly (*p* = 0.0289) greater in exudates of G.935 than M.26 rootstock. Right panel: relative chemotactic response of *Pratylenchus penetrans* to water agar, water agar + myo-inositol or neither as assessed in *in vitro* choice assays. Assay was conducted in triplicate with ten adult *P. penetrans* used in each replicate trial. The number of *P. penetrans* attracted to the myo-inositol treated agar plug was significantly (*p* < 0.03) lower than that toward the water agar alone or numbers migrating toward neither agar-based treatment.

The sugar alcohols sorbitol and myo-inositol (Figure 2) were detected at significantly higher concentrations in G.935 relative to M.26. Sorbitol significantly reduced bacterial populations in treated orchard soil and significantly altered composition of the microbiome while myo-inositol demonstrated repellant-like activity toward *P. penetrans*. These analyses imply that a diversity of rhizosphere metabolites possess the capacity to modulate activity of known ARD pathogens in the apple root zone and influence rootstock disease tolerance/susceptibility. Historical focus on a very few highly abundant metabolites has limited the view of root exudates as a potential contributor to ARD tolerance of apple rootstocks.

## Mechanisms of rootstock disease resistance

Developing functional host resistance to ARD is a major challenge due to the lack of consensus regarding disease causality, the reported variation in qualitative and quantitative composition of causal pathogens across orchard sites and the diversity of multitrophic interactions contributing to this complex disease. To date, the examination of active host resistance to ARD has focused on a limited number of genes, causal agents and rootstock genotypes. Multiple studies have revealed the upregulation of common biotic stress response genes in apple when cultivated in replant orchard soils (Zhu et al., 2016; Weiß et al., 2017a,b; Reim et al., 2020; Wang et al., 2021). However, the potential function of such genetic response in limiting host damage when exposed to the pathogen complex residing in replant soils is not certain as there was no attempt to associate these responses with host resistance. While a number of genes were uniformly upregulated among rootstock genotypes exposed to replant soils (Reim et al., 2020), these genes may largely serve as potential biomarkers for ARD but are unlikely to serve as targets for genetic improvement of apple rootstocks for resistance to the disease. For instance, upregulation of various biosynthesis and signaling genes important in plant defense responses, including phytoalexin production, was repeatedly observed (Shin et al., 2014; Weiß et al., 2017b; Wang et al., 2021) in apple rootstocks planted in ARD soil. The response, however, appears insufficient to defend against the pathogen complex encountered in replant soil (based upon the distribution of the response across genotypes differing in susceptibility to ARD). Overall, the trend in upregulation of these generalized defense response genes is similar in both highly susceptible and less susceptible apple rootstocks (Wang et al., 2021).

A plausible alternative is to initially employ sequential examination of rootstock germplasm for resistance to individual pathogens contributing to ARD. Although mechanistically undetermined, resistance to *Phytophthora cactorum* has been reported with certain Geneva series rootstocks. There are, however, notable differences in the reported level of resistance attributed to a given rootstock; G.935 and G.202 were deemed susceptible to *P. cactorum* in certain studies (Choi et al., 2021) but highly resistant in other evaluations.^1^

One of the few studies that examined host resistance to a replant pathogen which employed both phenotypic and genetic evaluation concerned an assessment of rootstock response to challenge with the oomycete *P. ultimum* (Shin et al., 2014, 2016). Progression of root necrosis and proliferation of hyphae along infected roots was delayed in resistant rootstock germplasm while necrosis and profuse hyphal growth was observed along roots of susceptible rootstock germplasm (Zhu et al., 2016, 2018). Transcriptome analysis revealed that the timeframe of molecular defense activation in response to *P. ultimum* challenge differed significantly between a resistant (G.935) and susceptible (Bud9) apple rootstock (Zhu et al., 2019). The transcriptome of the susceptible genotype Bud9 was more dramatically altered with a vast number of downregulated DEGs at 48 h post-inoculation (hpi). In contrast, the resistant G.935 rootstock exhibited a more modest disruption in the transcriptome in response to pathogen challenge with a majority of the DEGs being upregulated. Several genes involved in defense activation, including those encoding kinase receptors, jasmonic acid biosynthesis enzymes and jasmonic acid transcription factors, were upregulated earlier in G.935 roots relative to Bud9. It was hypothesized that the quicker and more consistent defense activation of an effective effector-triggered immunity response in the roots of G.935 limited the progression of necrosis incited by *P. ultimum* (Zhu et al., 2019). This premise does not appear to hold across the diversity of apple rootstock genotypes.

When cultivated in a known ARD orchard soil several biological processes related to defense were elevated at an earlier time point (48 hpi) in the disease susceptible rootstock M.26 relative to the tolerant rootstock G.210 (Wang et al., 2021). Subsequently, biological processes related to defense were elevated in G.210 relative to M.26 but not until the 7-day time point. As noted above, the tolerant/resistant rootstock G.210 initially appeared to allocate more resources toward root biomass production. Numerous DEGs potentially involved in biomass production were higher in G.210 relative to M.26. These included DEGs important in cellular glucan metabolic processes, the synthesis of hemicellulose and pectin (which are the components of newly formed cell walls), and genes involved in protein synthesis and DNA replication. Studies to date detail not only how rootstock cultivars varying in ARD susceptibility differ in their response to pathogen pressure but also indicate that the functional resistance response may vary across rootstocks demonstrating enhanced relative growth and yield performance on orchard replant sites. The apparent differential functional resistance response across rootstocks to the diversity of pathogens inciting ARD demonstrates the need for additional studies to identify the breadth of processes/genes that may confer host tolerance/resistance. Further elucidation of the genetic determinants of rootstock resistance to individual pathogens or the ARD pathogen complex will aide in the optimization of rootstock breeding programs pursuing host resistance as a disease management option.

Alternatively, examination of the genetic response in highly susceptible apple rootstock germplasm may be useful to breeding programs in the identification of markers that are suitable. For example, the systemic suppression of genes associated with primary metabolism, including glycolysis, was observed in roots of the highly susceptible rootstock Bud9 when exposed to *P. ultimum* (Zhu et al., 2019). A similar down-regulation of genes involved in primary metabolism was detected in roots of the ARD susceptible rootstock M.26, relative to the tolerant rootstock G.210 (Wang et al., 2021). When employed in analysis using a standard “resistant” rootstock and previously uncharacterized plant material, such a comparative assessment in the activity of either an individual or a panel of genes may be a suitable factor for removal of specific genotypes during the screening of breeding generated populations.

## Functionality of soil amendment-rootstock genotype integration

In order to assemble multiple disease control options into an effective integrated disease management protocol, functional modes of action of independent treatments must be ascertained. Such knowledge is required to both determine appropriate means of optimizing pathogen suppression and also to avoid combinations in time or practice that diminish treatment efficacy. Development of an integrated approach to the management of ARD has generally been lacking in terms of defining modes of action determining disease control as well as optimizing efficacy beyond the “try and see what happens” approach. A broad spectrum of soil amendments, including composts (Wilson et al., 2004; Yao et al., 2006; Van Schoor et al., 2008; Braun et al., 2010), fertilization programs (Traquiar, 1984; Wilson et al., 2004; Cavael et al., 2021) and use of green manure or cover crops (Edwards et al., 1994; Merwin, 1995; Mazzola and Mullinix, 2005; Yim et al., 2017; Kanfra et al., 2021) have been evaluated for the ability to ameliorate replant disease symptoms. In large part, these soil amendment strategies were unsuccessful in managing ARD (Wilson et al., 2004; Yao et al., 2006; Van Schoor et al., 2008) although a short-term benefit in terms of vegetative growth was, at times, observed. Rumberger et al. (2004) observed no growth benefits of pre-plant compost amendment in apple cultivated on a replant site irrespective of rootstock genotype. In addition, although the rhizosphere bacterial community differed between susceptible (M.7 and M.26) and tolerant (G.30 and G.210) rootstocks, no difference was observed between the control and compost treatment for any rootstock (Rumberger et al., 2004). A significant limitation to extending use of these practices was the failure to assess treatment effect on the pathogen complex that incites ARD. The short-term benefits to tree growth developed as a result of enhanced soil fertility and moisture conditions rather than pathogen suppression and disease control.

Alternative soil amendment-based strategies, including pre-plant application of Brassicaceae seed meal (BSM) and use of the process termed anaerobic soil disinfestation (ASD), have demonstrated active control of ARD. In assessments of findings from previous controlled environment and field-based studies, it was either suggested that the efficacy of BSM was site and soil dependent (Hanschen and Winkelmann, 2020), that it was not a reliable and transferable management strategy across different locations (Cavael et al., 2021) or that it was ineffective against oomycetes (Deakin et al., 2019) and was not, therefore, a suitable treatment for the control of ARD. Such statements were reasonably based upon early studies that examined a number of BSMs that were applied individually (Mazzola and Mullinix, 2005) prior to determining their efficacy and modes of action in suppressing the diverse spectrum of ARD pathogens (Mazzola et al., 2007). However, those previous statements have been invalidated based upon subsequent studies employing a novel BSM formulation (Mazzola and Brown, 2010). Use of this optimized formulation produced maximum efficacy in control of ARD at all commercial scale orchard trials across numerous sites varying in soil and geographic setting (Mazzola et al., 2015; Wang and Mazzola, 2019a; Dupont et al., 2021). That is, the level of disease control along with apple growth and yield was equivalent to or exceeded that attained in response pre-plant soil fumigation (the commercially employed ARD control strategy).

Disease control in response to Brassicaceae residues applied as a soil amendment has generally been attributed to a process termed “biofumigation” (Matthiessen and Kirkegaard, 2006; Hanschen and Winkelmann, 2020). This designation implies its sole function is a chemical mode of action generally ascribed to chemistries derived from the hydrolysis of plant glucosinolates (e.g., isothiocyanates). Unfortunately, a focus on the fumigant-like potential of BSM amendments has led to misguided application of these treatments. Previous reports of disease control obtained long after active chemistries were evacuated from the soil system (Lewis and Papavizas, 1971; Cohen and Mazzola, 2006) or when Brassicaceae residues that do not yield antimicrobial chemistries were employed (Manici et al., 1997; Potter et al., 1998; Mazzola et al., 2001; Cohen et al., 2005) indicated that non-chemical mechanisms contribute to disease suppression. Additional studies demonstrated that a biologically intact soil system along with a functionally transformed rhizosphere microbiome is required for the effective suppression of multiple plant pathogens in response to BSM amendments (Cohen and Mazzola, 2006; Mazzola et al., 2007; Weerakoon et al., 2012).

Similarly, suppression of soilborne pathogens in response to anaerobic soil disinfestation has been attributed to various active chemistries generated during the anaerobic phase of the treatment process (Momma et al., 2006; Runia et al., 2014; Hewavitharana et al., 2015; Rosskopf et al., 2020). However, as in the case of BSM amendment, the soil/rhizosphere microbiome that developed in response to ASD was essential to the generation of conditions and/or mechanisms leading to pathogen suppression and disease control (Hewavitharana and Mazzola, 2016). Thus, use of amendment-based strategies must consider the various attributes of the orchard system that influence composition and function of soil microbial resources. This includes attention to appropriate rootstock genotype which not only varies in ARD susceptibility but also has a significant role in shaping composition of the soil, rhizosphere and endophytic microbiome (Rumberger et al., 2004; Deakin et al., 2019; Wang and Mazzola, 2019b; Van Horn et al., 2021).

Efficacy of BSM in control of ARD was influenced by rootstock genotype in multiple field trials (Mazzola et al., 2015; Wang and Mazzola, 2019b). Heightened treatment efficacy was commonly observed with BSM soil amendment when used in conjunction with Geneva rather than Malling series apple rootstocks. The differential rootstock performance was characterized by increased growth and yield, efficacy attained at lower amendment rates, diminished sensitivity to herbicidal activity and reduced pathogen densities when the treatment employed a Geneva rootstock (Mazzola et al., 2015; Wang and Mazzola, 2019b). For example, Gala/M.26 trees exhibited significant mortality when BSM was applied at the highest rate (6.6 t ha^–1^) immediately prior to spring planting while no phytotoxicity was observed for Gala/G.41 trees (Wang and Mazzola, 2019b). Although Gala/G.11 and Gala/M.9 trees planted in non-treated ARD soil grew similarly, Gala/G.11 generated significantly higher yields than Gala/M.9 when cultivated in *B. juncea/S. alba* seed meal amended soil (Mazzola et al., 2015). This differential yield response in BSM-treated soil was associated with significantly lower populations of *P. penetrans* and *Pythium* spp. recovered from G.11 roots relative to M.9 at 2 years post-planting. Similarly, when BSM was applied immediately prior to planting in spring at 4.4 or 6.6 t ha^–1^, Gala/G.41 reached yields that were significantly higher than Gala/M.26 which was associated with significantly lower densities of *P. penetrans* in G.41 than M.26 roots (Wang and Mazzola, 2019b).

Pre-plant BSM soil treatment provides disease control at levels equivalent to or better than pre-plant soil fumigation regardless of rootstock genotype. However, the superior performance of Gala/G.41 in BSM-treated soil noted above was associated with a root and rhizosphere microbiome that was more distinct from the no treatment control treatment than the corresponding microbiome from Gala/M.26 (Wang and Mazzola, 2019b). Although the rhizosphere microbiome from Gala/G.41 and Gala/M.26 were highly similar in the non-treated soil, microbiome composition in the rhizospheres of the two rootstock genotypes became more divergent when cultivated in BSM-treated soil. Dissimilarity in microbiome composition increased with increasing seed meal amendment rates (Wang and Mazzola, 2019b). Differential recruitment of plant beneficial microbes to the rhizospheres of susceptible (M.26) vs. tolerant (G.210) rootstocks and/or the differential effects on initiation of host plant defense responses may have led to a greater protective effect of the BSM treatment on G.210 (Wang et al., 2021). When cultivated in BSM amended ARD soil, both rootstocks exhibited differential gene expression relative to corresponding non-treated control soil. However, as noted above, the dynamics of gene expression indicated that the BSM-amended soil system altered the trajectory of the root transcriptome in a genotype-specific manner. Altered gene expression was temporally associated with changes in rhizosphere microbiome density and composition in the BSM-treated soil (Wang et al., 2021).

Rootstock genotype has also demonstrated variable effects on the efficacy of ASD to control apple replant disease. In greenhouse and field trials that utilized rootstock liners as the planting material, ASD significantly enhanced tree growth relative to non-treated ARD orchard soil irrespective of genotype (Hewavitharana and Mazzola, 2020). Disease susceptible (M.9) and tolerant (G.41 and G.935) rootstock genotypes performed similarly in terms of increased tree growth in response to ASD in a manner equivalent to that achieved in fumigated soil. In the same study, ASD significantly reduced the quantity of *R. solani* AG-5 DNA detected in apple roots relative to the no treatment control but no significant difference was observed between rootstock genotypes. These findings suggested that ASD could be used successfully for the management of ARD as an independent treatment across rootstock genotypes varying in disease tolerance. However, in a subsequent field trial that employed grafted trees, efficacy of ASD in terms of improved tree growth was rootstock genotype-dependent (Dupont et al., 2021). Although ASD uniformly improved tree growth in a manner similar to that attained in response to pre-plant soil fumigation, growth of Cosmic Crisp/G.41 (tolerant) was significantly greater than Cosmic Crisp/M.9 (susceptible) in ASD treated soil (Dupont et al., 2021). Trees exhibiting increased growth in both ASD and fumigated soil possessed significantly lower root densities of *P. penetrans* relative to the no treatment control. In addition, the improved tree growth observed in response to ASD across three orchard sites was consistently associated with significant changes in the rhizosphere and soil microbiome, regardless of rootstock utilized. In general, ASD resulted in increased abundance of Actinobacteria, Bacteroidetes and Firmicutes. Among the Firmicutes those exhibiting increased relative abundance were primarily members of the Bacillales and Clostridiales (Dupont et al., 2021). These groups are known to function in the production of antimicrobial metabolites derived during ASD which contribute to the suppression of soilborne pathogens (Hewavitharana et al., 2019).

Findings from studies concerning the integration of rootstock genotype with amendment-based disease control strategies demonstrate the breadth of knowledge required to attain optimal efficacy. In the two systems explored above, there exists a greater understanding of factors that regulate the interaction between rootstock genotype and BSM soil amendment than the rootstock genotype-ASD interaction. While apparent differences in growth response and disease control attained between rootstocks varying in disease tolerance was consistent with BSM treatment across soils and experiments, a greater level of variability in the response for a given rootstock genotype was observed across ASD trials. In part, the consistency in comparative efficacy observed across trials for ARD tolerant or susceptible rootstocks observed with BSM amendment was associated with the timing of sequential changes in the rhizosphere microbiome supported by different rootstock genotypes. These dissimilarities could have differentially affected initial pathogen suppression as well as initiation of host defense mechanisms in the tolerant and susceptible rootstocks. It is clear that efficiency of BSM amendment for control of ARD will be elevated when applied in concert with a tolerant apple rootstock genotype (Wang and Mazzola, 2019a).

## Priorities for future research and development

Management of apple replant disease has traditionally relied upon the availability and use of broad-spectrum soil fumigants prior to orchard replanting. Establishing environmentally sustainable and effective measures for control of the disease will require formulation of novel approaches that consider the entirety of the orchard ecosystem. The plant-induced transition of the soil microbiome to yield conditions conducive to ARD demonstrates the need for a comprehensive understanding of the structuring forces that shape microbial community composition and the functional dynamics underlying the evolution or suppression of the disease. Such knowledge will facilitate the development of sustainable disease control measures including those employing amendment-based management approaches designed to harness the potential of the indigenous microbiome. Due to the highly inter-connected nature of the plant genotype and the plant microbiome, harnessing the plant’s genetic potential to manipulate root-associated microbiomes (independently or in combination with amendment-based strategies) is an important and integral step toward improving productivity in orchard ecosystems. That said, apple replant disease tolerance is a very complex root trait which is difficult to phenotype; susceptibility or tolerance to different ARD pathogens may differ mechanistically among rootstock genotypes.

So far, what has been learned about *why* and *how* apple rootstock genotype influences ARD defense has been attained through multiple research approaches (metabolomic, microbiome, and transcriptomic-based studies), all of which we have attempted to highlight in this review. In conjunction with such efforts, it is essential to develop screening programs which assess the genetic potential of different rootstocks to stimulate or inhibit *multiple* components of the pathogen complex (individually and collectively). Such a strategy may prove invaluable if replant pathogen consortia are ultimately confirmed to differ with the geographic region (although this is yet to be proven). Finally, we know much more about the apple rhizosphere than endophytic microbiome. In apple, research on the endophytic microbiome has only scratched the surface, with most efforts to characterize this group limited to a few rootstocks in a single soil system. This, therefore, represents another important avenue for future study. An understanding of the complex interactions between apple roots, ARD pathogens and beneficial microorganisms in the rhizosphere and endosphere will require new conceptual approaches to investigation that must integrate cutting-edge omics-based approaches with proof-of-concept studies that employ the biological entities identified. As we enter an era of increasing challenges to the function of agricultural ecosystems across the planet (both regulatory and environmental), these are directions of research which will aide scientists and growers in addressing the questions and finding viable solutions.

## Author contributions

All authors listed have made a substantial, direct, and intellectual contribution to the work and approved it for publication.

## Funding

Funding for this research originated from the United States Department of Agriculture-Agricultural Research Service; National Program 303: Plant Diseases; Project # 2094–21220-003-000-D.

## Conflict of interest

The authors declare that the research was conducted in the absence of any commercial or financial relationships that could be construed as a potential conflict of interest.

## Publisher’s note

All claims expressed in this article are solely those of the authors and do not necessarily represent those of their affiliated organizations, or those of the publisher, the editors and the reviewers. Any product that may be evaluated in this article, or claim that may be made by its manufacturer, is not guaranteed or endorsed by the publisher.

## Footnotes

^1^ https://extension.psu.edu/apple-rootstocks-capabilities-and-limitations
